# GerAB residues predicted to interfere with water passage based on steered molecular dynamics are key to germinosome functionality

**DOI:** 10.3389/fmicb.2025.1656964

**Published:** 2025-09-24

**Authors:** Longjiao Chen, Houdijn Beekhuis, Christina van den Bosch, Gianni Vinay, George Korza, Jocelyne Vreede, Peter Setlow, Stanley Brul

**Affiliations:** ^1^Swammerdam Institute for Life Sciences, University of Amsterdam, Amsterdam, Netherlands; ^2^Van ‘t Hoff Institute for Molecular Sciences, University of Amsterdam, Amsterdam, Netherlands; ^3^Department of Molecular Biology and Biophysics, UConn Health, Farmington, CT, United States

**Keywords:** bacterial spore, spore germination, molecular dynamics simulation, APC transporter, water channel

## Abstract

Some *Bacillales* and *Clostridiales* bacteria form spores in unfavorable environments. These spores are dormant but can rapidly resume metabolism in germination. This process can be initiated by a variety of low molecular weight nutrients termed germinants. Structural modeling and mutagenesis studies showed that GerAB, an inner membrane (IM) protein of the *Bacillus subtilis* spore germinant receptor (GR) GerA, is involved in L-alanine-initiated spore germination. A previous molecular simulation study also suggested there is a water channel in GerAB. In the current work, Steered Molecular Dynamics (SMD) simulations were employed to force a single water molecule through GerAB, identifying three key amino acid residues, Y97, L199 and F342, that interfere with water passage. When these residues were altered to alanine, L-alanine germination no longer occurred in spores with L199A, F342A and triA (Y97A, L199A and F342A triple mutant), while Y97A mutant spores germinated ∼61%. Additionally, except for Y97A, all other mutants showed compromised germination triggered by the AGFK mixture (L-asparagine, D-glucose, D-fructose and K^+^ ion). Western blotting found reduced levels of the GerA GR in the Y97A mutant, and an absence of the GerA GR in all other mutants. This proves that all three identified residues are crucial to the structural integrity of the GerA germinant receptor and also suggests they are essential for the formation of a fully functional GR complex, the germinosome.

## 1 Introduction

Several species within the *Bacillales* and *Clostridiales* bacterial orders can form dormant spores in harsh environments. These spores are metabolically inert, extremely resistant to a variety of harsh treatments, and dormancy can last for decades. However, cells derived from some of these spores can cause food spoilage, infectious diseases, intoxications and foodborne illnesses (Christie and Setlow, 2020). This is especially true for spores of *Bacillus anthracis*, *Clostridiodes difficile* and *Bacillus cereus*. Consequently, the biology of spore-forming bacteria has been investigated for decades. A major feature of dormant spores is their low core water content, 25%–45% of wet weight in spores of the model organism *Bacillus subtilis*, compared to ∼80% of wet weight in its growing cells. However, how water is taken up into the dormant spore core is not clear, as *B. subtilis* lacks known water pores such as aquaporins (Sunde et al., 2009; Knudsen et al., 2015).

The loss of spore dormancy and resumption of its metabolism and cell-like structure requires spore germination. Small molecule nutrients, termed germinants, including specific monosaccharides, monovalent cations, and amino acids, can trigger spore germination with no germinant catabolism involved (Moir et al., 2002). Germinant receptors (GRs) play a crucial role in kick-starting germination. There are three GRs in the model organism *B. subtilis*, GerA, GerB, and GerK. Each GR is composed of three subunits, A, B, and C, and requires the presence of all three subunits to function (Moir et al., 2002; Christie and Setlow, 2020). GRs and GerD protein colocalize in a cluster termed the germinosome in the spore inner membrane (IM). Even though the detailed germinosome structure is unknown, its structural stability is necessary for rapid and cooperative responses to nutrients through GRs (Christie and Setlow, 2020). The B subunit of GerA, GerAB, is a transmembrane protein belonging to the amino acid-polyamine-organocation (APC) transporter family that mediates L-alanine-triggered germination (Zuberi et al., 1987; Yi and Setlow, 2010; Artzi et al., 2021). Similar function and organization were identified for GerB and GerK except these two collectively respond to the AGFK mixture (L-asparagine, D-glucose, D-fructose and K^+^ ions) (Cabrera-Martinez et al., 2003) instead. Many attempts have been made to fully understand the structure and function of GRs. Recently, GerAB has been shown to be the ligand sensing subunit (Artzi et al., 2021) using structure prediction and mutagenesis. With Molecular Dynamics (MD) simulations, Blinker et al. (2021) identified a putative water channel in GerAB (Smart et al., 1996). A recent study by Gao et al. (2023) combined AlphaFold (Jumper et al., 2021) structure prediction and mutagenesis, showing that GerA forms an oligomeric membrane channel which releases monovalent cations upon sensing L-alanine. However, to date, the function and mechanism of the putative water channel in GerAB and its role in spore water intake during germination had neither been fully elucidated nor experimentally verified.

**FIGURE 1:**
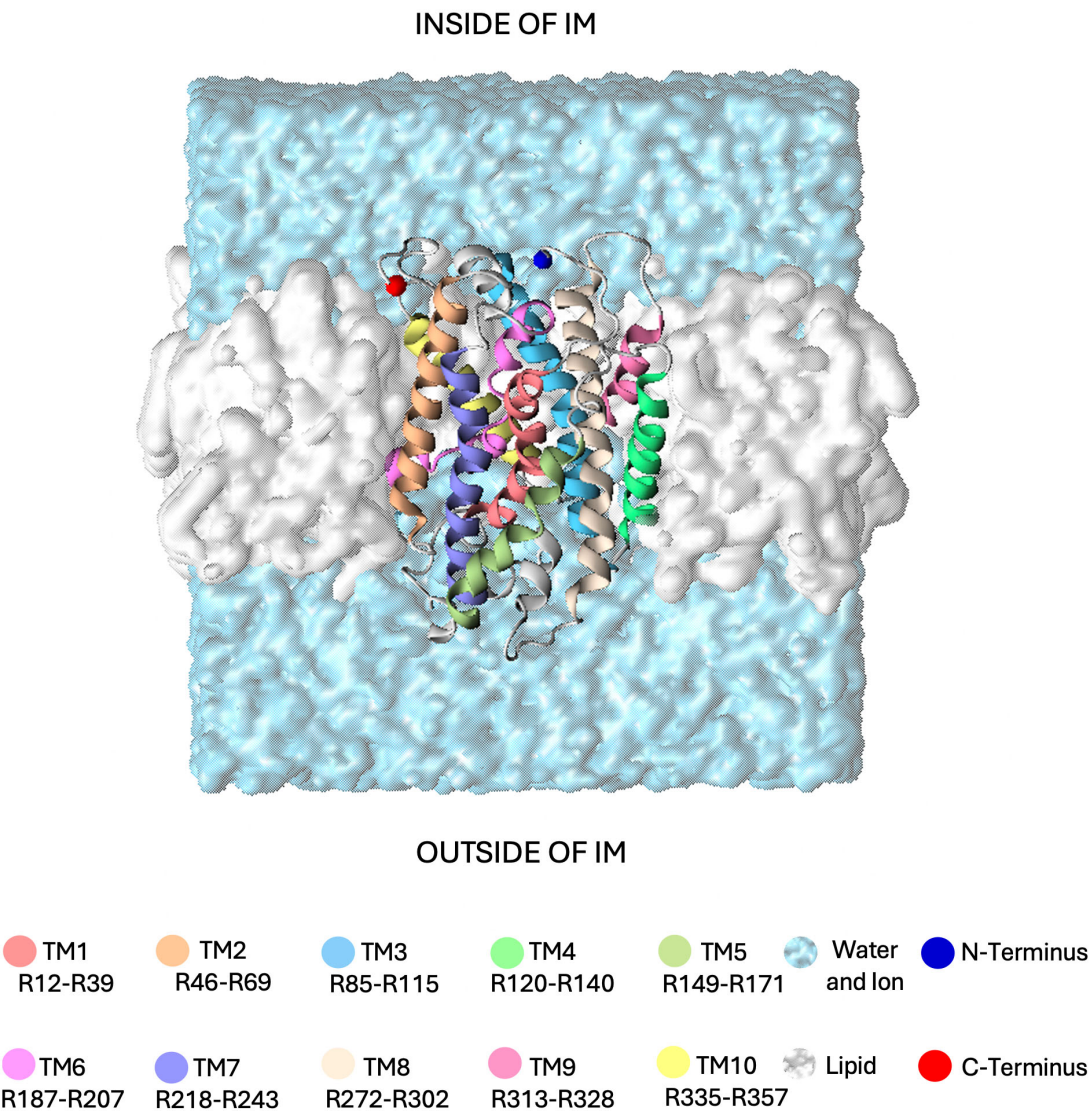
Simulation system used in this study. The GerAB protein is embedded in a lipid bilayer membrane (shown as a white surface), with its N- and C-termini oriented toward the interior of the spore’s inner membrane (IM). The protein is displayed as a ribbon, with the 10 transmembrane regions (TMs) individually color-coded. Residues assigned to each TM follow the prediction by Blinker et al. (2021). The surrounding water, containing 20 mM KCl, is represented as a light blue surface.

In this study, building on the observation of GerAB internal hydration and the consistent presence of a water channel with a 0.1–0.3 nm radius in all-atom MD simulations (Smart et al., 1996; Blinker et al., 2021), we noted that spontaneous water crossing events hardly occurred within 100 ns. To model water crossing within the time span accessible to all-atom MD simulations, we employed Steered Molecular Dynamics (SMD) to pull a water molecule through the protein. In our approach, a single water molecule was pulled from the extracellular side to the intracellular side of GerAB to explore potential water crossing pathways. In these simulations, the orientation of GerAB was assumed to have its termini located on the inside of the spore (Figure 1), similar to other GR structural studies in *B. subtilis* (Gao et al., 2023) and proven in *B. anthrax* by using a GFP terminal fusion (Wilson et al., 2012). This setup allowed us to assess mechanisms of water passing through GerAB, as the first step to understand water crossing. However, due to limitations of the chosen pulling coordinate, our current SMD setup is not able to provide an estimate of the potential of mean force. Note that the SMD simulations are presented here as an exploration of potential water crossing mechanisms, with the aim of identifying crucial residues in GerAB.

By coupling SMD with mutagenesis and germination assays, this study identified Y97, L199 and F342 as potential blocks for water passage. We successfully constructed Y97A, L199A and F342A single mutant and triA (Y97A, L199A and F342A) triple mutant spores. A Western Blot confirmed that Y97A successfully assembled GerA, albeit at a reduced level. Moreover, we observed that spores without a functional GerA exhibited decreased function of GerB and GerK GRs. Collectively, these results showed that L199 and F342 are crucial for the structural assembly of GerA, while Y97 is important in germinosome function.

## 2 Results

### 2.1 Key residue identification

**FIGURE 2:**
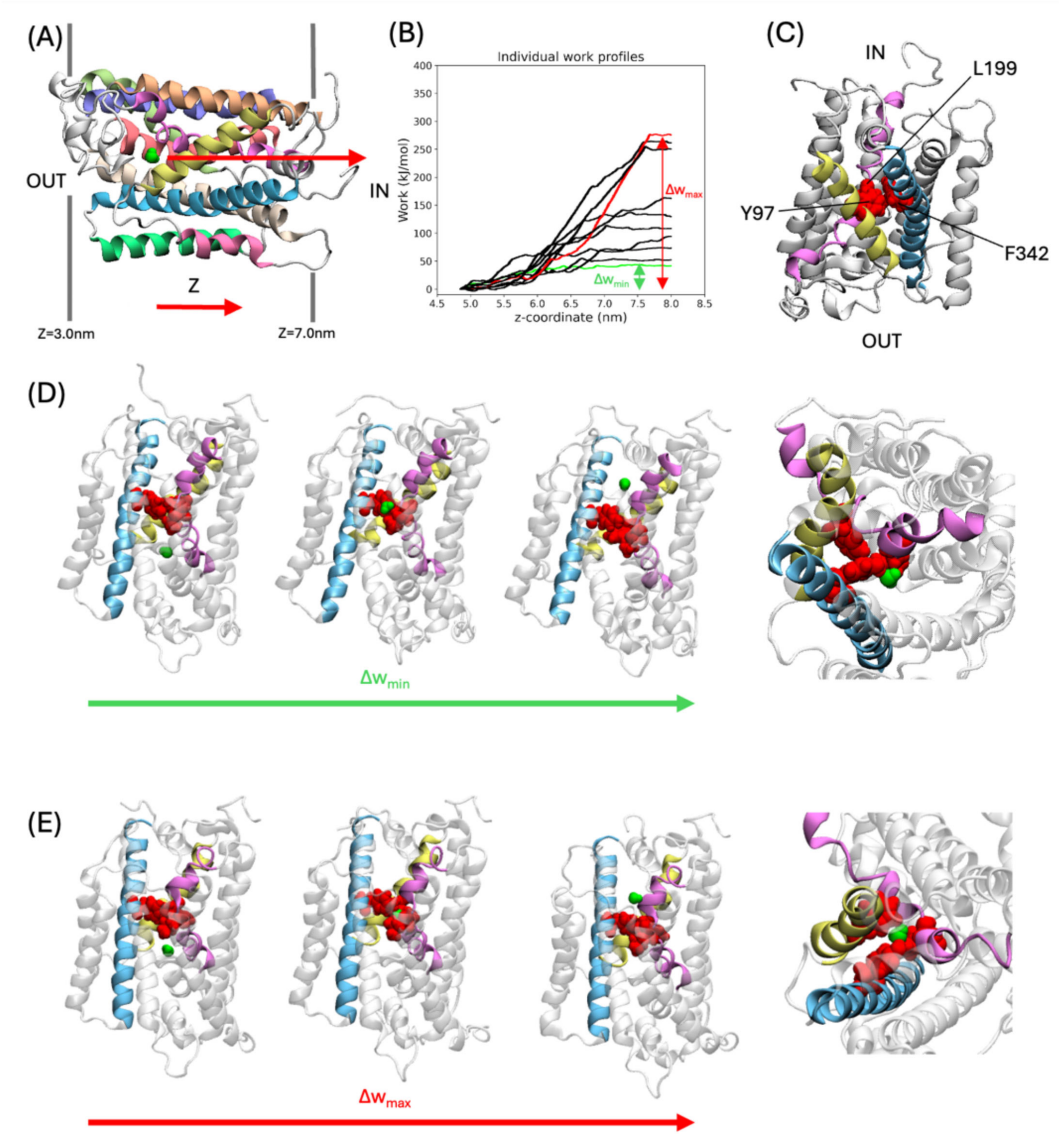
SMD Simulations of water molecules pulled through GerAB. **(A)** Example of the starting frame for the SMD simulations. The water molecule which is pulled is shown in a green space filling representation. The arrow indicates the pulling direction, from the outside of the spore IM to the inside. Dark gray lines flanking the protein indicate the membrane. **(B)** Work profiles of the SMD simulations along the z-axis. The work profiles Δw_max_ and Δw_min_ are shown in red and green, respectively. **(C)** A triad of Tyr97, Leu199, and Phe342 (all in red space filling representation) are located on TM3, TM6, and TM10, respectively. Only TMs with the triad are colored, according to the coloring scheme in Figure 1. Protein regions not part of the triad is shown in white. **(D,E)**, Water molecule passing the triad in the SMD simulation with Δw_min_ and Δw_max_, respectively. From left to right: snapshots of simulation for water oxygen at *z* = 4.84 nm, 5.89 nm, 6.65 nm (for Δw_min_), and *z* = 5.07 nm, 6.21 nm, 7.84 nm (for Δw_max_), showing the water before, during, and after passing the triad, followed by a closeup of the triad with the water molecule during the water molecule passing the triad. The closeup is shown from the inside of the spore.

In the SMD simulations, water molecules were pulled through GerAB from the outside of the spore IM to the inside, as indicated in Figure 2A. For each individual SMD run, the cumulative work as a function of the z coordinate (perpendicular to the plane of the membrane) was calculated. For all runs, the exerted work increases at *z* = 5 nm, until *z* = 7.5 nm. This means that the water molecule does not spontaneously go through the protein at the pulling speed. At *z* > 7.5 nm, the cumulative works stays approximately constant, which means that no additional work is required to pull the water molecule through bulk water after it has exited the protein (Figure 2B). This observation shows that the SMD does not suffer from artifacts caused by pulling the water too fast, as pulling the water molecule through bulk water happens at a velocity typical for water diffusion at 298 K (Maginn, 2007). This indicates that the pulling speed is sufficiently slow.

The pulling work profiles all have a similar shape but differ in when the plateau is reached. To quantify this variation, differences in the cumulative work are defined as Δw = w_end_ – w_start_. Δw ranged from Δw_min_ = 37 kJ/mol to Δw_max_ = 293 kJ/mol (Figure 2B). The Δw values show a large variation and are reported here to indicate that there are different ways in which the water molecule can go through the protein when pulled. In all simulations, a group of three amino acids with bulky side chains termed the triad, consisting of Y97 (located on TM3), L199 (located on TM6), and F342 (located on TM10) (Figure 2C), blocked the movement of the water molecule along the z-axis. To move through GerAB across the blockade formed by the triad, the water molecule passed on the side of L199 and F342, located between TM6 and TM10, and further away from Y97 in the run with Δw_*min*_ (Figure 2D). In contrast, in all other SMD simulation trajectories, the water molecule went through the center of the three bulky side chains forming the triad, as exemplified by run Δw_*max*_ (Figure 2E). A previous study (Blinker et al., 2021) using MD simulations and HOLE analysis (Smart et al., 1996) predicted a water channel formed by TM1, 2, 3, 6, and 8. In this study, we identified Y97 and L199 located on TM3 and TM6, respectively. We also identified F342 on TM10. Since the bulky side chain triad interfered with water molecule passage in all the SMD simulations trajectories, we checked if they are conserved in other *Bacillus* spore-forming species. With CLUSTAL Omega (Madeira et al., 2024), we aligned the amino acid sequences of GerAB with those of GerBB, GerKB from *B. subtilis*, GerVB from *Bacillus megatarium* QM B1551 and GerRB from *B. cereus* ATCC14579. This sequence alignment indicated that Y97 is fully conserved, while F342 and L199 are not conserved in the three germinant receptor subunits (Supplementary Figure 1). Therefore, Y97 might have a functional role in GRs of spore formers beyond *B. subtilis*. To verify the function of the triad *in vivo*, the triad residues have been mutated to alanine to test the function of the bulky side chains in the germination behavior of *B. subtilis* spores.

### 2.2 Y97, L199 and F342 are important in the functionality of the germinosome

**FIGURE 3:**
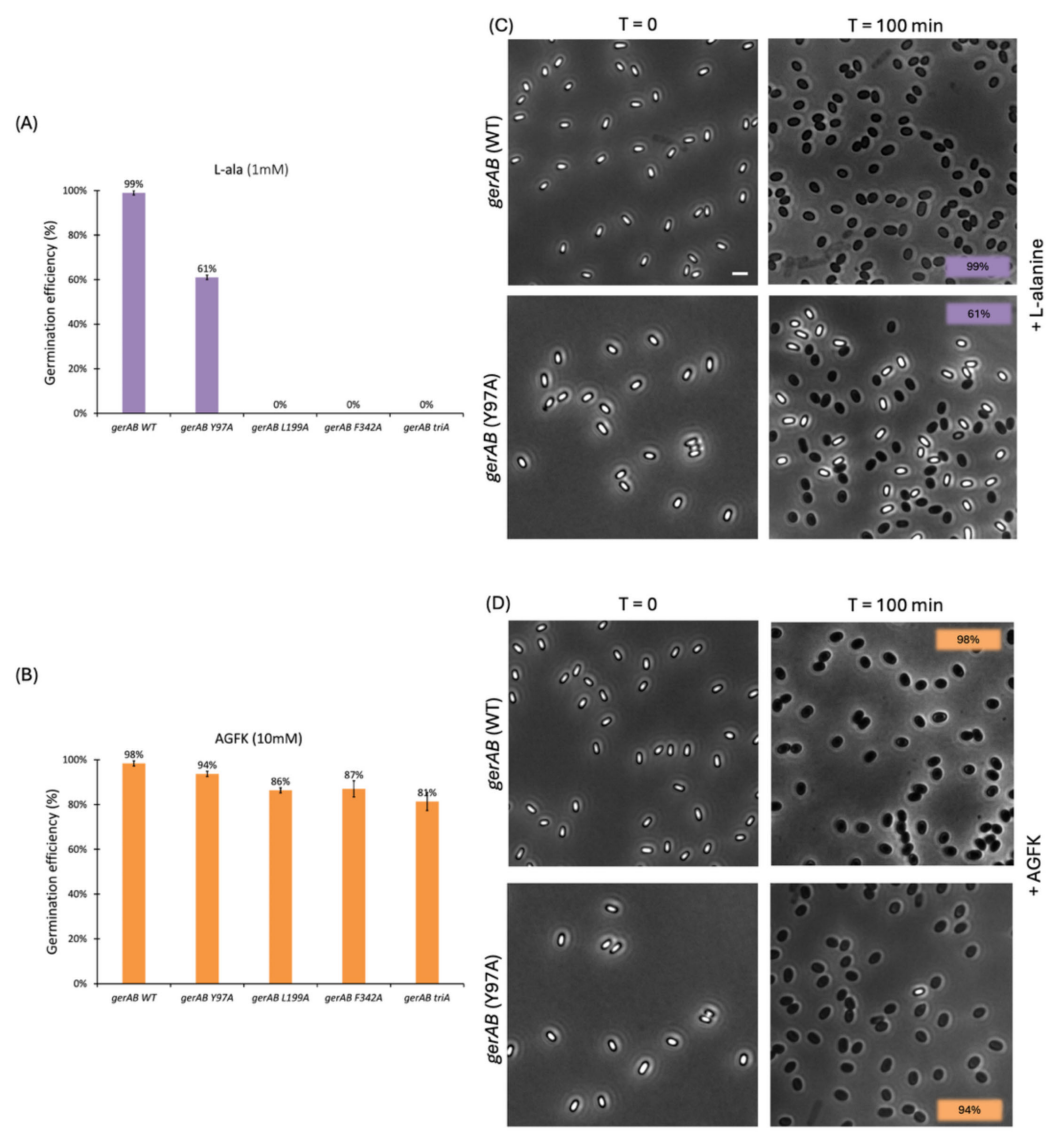
Single spore germination assay of wt and different spore variants by phase contrast microscopy. Germination efficiency of spores with different GerAB variants with L-alanine **(A)** or AGFK **(B).** Wild type spores’ germination was close to 100% with both germinants. In responds to L-alanine, only Y97A germinates 61% while other mutants are unable to germinate. In respond to AGFK, Y97A spores exhibits the highest spore viability among all four mutants. **(C,D)** Representative phase-contrast microscopy images of wt and Y97A spores before and after a 100 min incubation with L-alanine or AGFK, respectively. Dormant spores are phase bright while germinates spores are phase dark under the current microscopy condition. Scale bar, 2 μm. Error bars in Panel **(A,B)** indicate mean ± SD of three technical replicates. Uncropped microscopy images are presented in Supplementary Figure 2.

**FIGURE 4:**
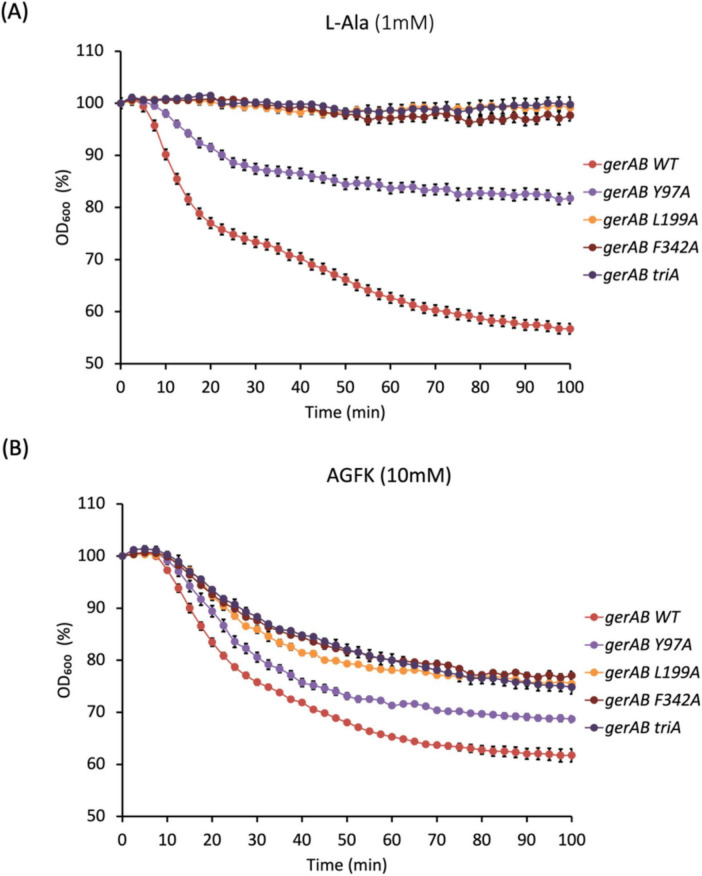
Spore germination measured by OD_600_ drop triggered by 1 mM L-alanine **(A)** or 10 mM AGFK **(B)**. Y97A mutant spores showed partial germination compared to wt spores in response to L-alanine. L199A, F342A and triA mutant spores failed to germinate in the presence of L-alanine. All mutants showed compromised germination with AGFK, although less so in Y97A mutant. Error bars indicate ± SD of three technical replicates.

Four mutant strains of *B. subtilis* PY79 were successfully constructed, Y97A, L199A, F342A, and triA (Y97A, L199A and F342A triple mutant). Differences in germination efficiency of each strain with L-alanine or AGFK shows that each triad residue has an important role in both L-alanine mediated germination and AGFK initiated germination due to the reduced ability of mutant spores to respond to both types of germinants compared to wt (wild-type) spores. In the presence of L-alanine, only Y97A mutant spores germinated, with a germination efficiency of 61%, while the other mutants did not respond to L-alanine (Figures 3A, C). A similar pattern occurred when measuring germination by the OD_600_ drop where Y97A spores showed a partial OD_600_ drop (∼85%) compared to that of wt spores (∼60%), while L199A, F342A and triA mutants exhibited no OD_600_ drop under same experimental conditions (Figure 4A). All mutants responded to AGFK with slightly slower kinetics compared to wt spores. Note that a lower OD_600_ drop indicates a lower germination efficiency, albeit with different levels of the different mutant spores (Figure 4B). This is in agreement with single-spore germination efficiency assays, which revealed a drop around 10% of germination efficiency in L199A, F342A and triA mutant spores (Figure 4A). Notably, the OD_600_ drop, and the germination efficiency drop with Y97A spores are both less than that of the other mutant spores.

**FIGURE 5:**
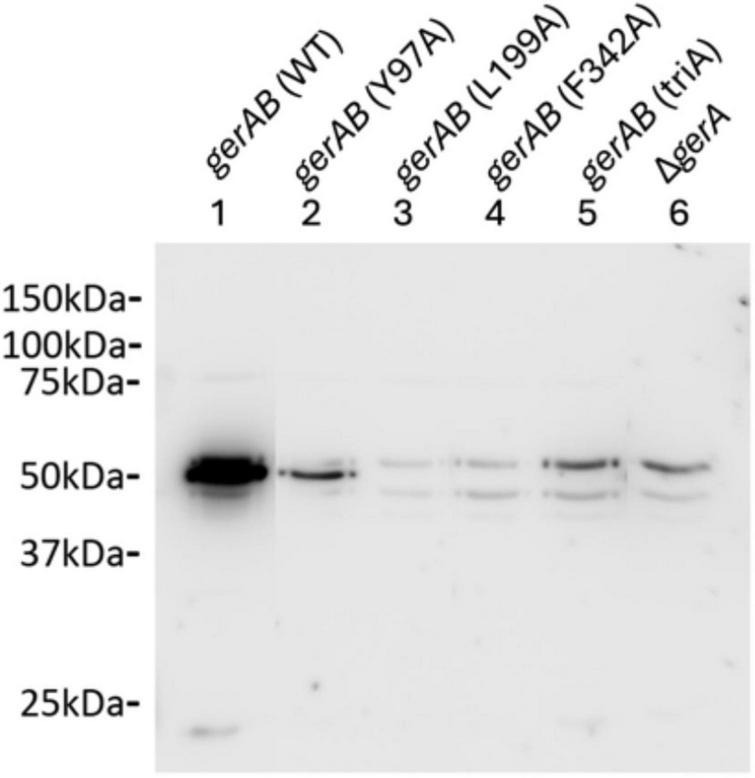
Western blot of *B. subtilis* PY79 wt and mutant spore proteins with anti-GerAA antibody. Both *gerAB* (wt) and *gerAB* (Y97A) sample show a clear band with the GerAA antibody at ∼50 kDa, while the other mutants showed no GerAA band. However, *gerAB* (L199A), *gerAB* (F342A) and *gerAB* (triA) spores exhibited only the same bands as the Δ*gerA* GerAA deletion control sample. All samples were taken from the same blot; irrelevant lanes were removed. Uncropped blot image is presented in Supplementary Figure 3.

To determine whether the mutant GerAB proteins were incorporated in the spores, we relied on the finding that GR stability depends on a strict 1:1:1 stoichiometry of the A, B and C subunits, and that the existence of GerAA and GerAC in spores depend on the stability of GerAB. This means that the number of GerAA proteins in spores is directly related to the number of GerAB proteins and the GerA complexes. Absence or reduction of GerAA levels in the mutants is a reliable marker for failure of assembling the complex (Cooper and Moir, 2011; Mongkolthanaruk et al., 2011). We therefore performed western blot analysis on wt and mutant spore lysis using a purified GerAA antibody. Only wt and Y97A mutant spores exhibited GerAA signals, although the level in the Y97A mutant is lower than that in wt spores (Figure 5). Since assembly of the GerA GR requires all three subunits, this means that the lack of response to L-alanine of L199A, F342A and triA mutant spores was due to the absence of whole GerA GR (Cooper and Moir, 2011). That is to say GerAB Y97A is produced and assembled into a complex with GerAA and GerAC, albeit the lower level may indicate its lower stability. At the same time, GerAB L199A, GerAB F342A and GerAB triA are either unstable or unable to form a complex with GerAA and GerAC proteins. These findings indicate that residues Y97, L199, and F342 are all critical for maintaining the structural stability of GerAB. Among them, L199 and F342 appear to be even more essential than Y97 for GerAB structure, as their mutation completely abolished the formation of the GerA germinant receptor complex.

To explore the structural aspects of the triad residues, we performed protein contact analyses on the ten starting structures of the SMD simulations, listed in Supplementary Table 1 in the Supporting Information. In particular, F342 has at least one π–π stacking interactions in all starting structures with F55, Y97, F198 or F346. While Y97 interacts with only F342 in five starting structures via π–π stacking. Counting the number of contacts, with a contact defined as two residues within 7Å. L199 showed the highest total number of contacts in seven of the starting structures (Supplementary Table 1). These results suggest that L199 and F342 engage in more interactions within GerAB, and their mutation has a stronger destabilizing effect on the protein compared to Y97. However, with respect to the original hypothesis of this study, the inability of the L199A, F342A, and triA mutants to form the GerA complex prevents us from directly testing whether L199 and F342 function as a barrier to water crossing. In contrast, the Y97A mutant was still able to form the GerA complex and exhibited reduced germination efficiency. Yet, it remains unclear whether this reduction is due to lower numbers of GerA complexes or a dysfunctional water channel.

**FIGURE 6:**
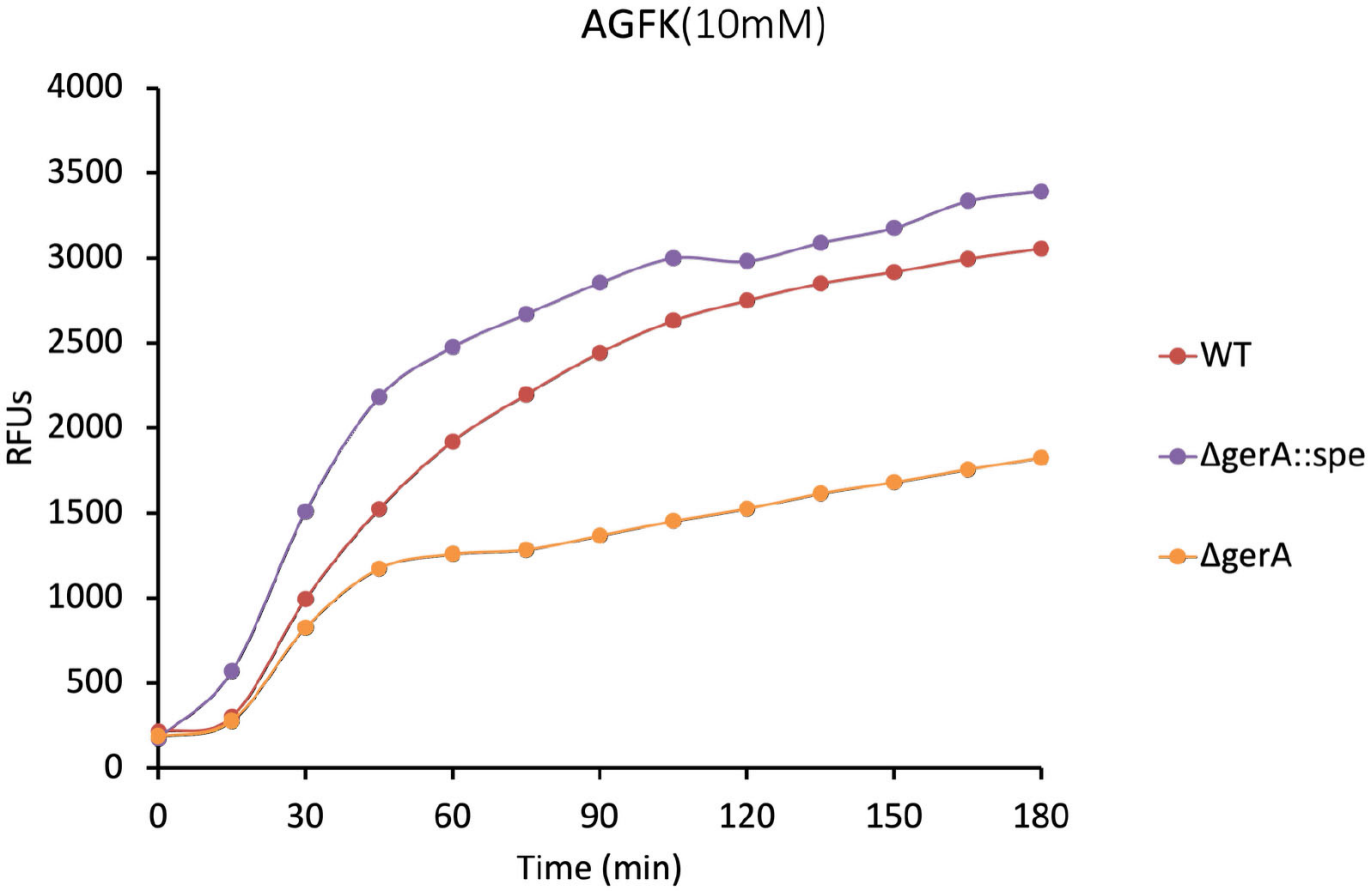
Germination of Δ*gerA* strain compared to wt based on DPA release. The Δ*gerA* strain showed partial AGFK germination compared to the wt strain. Since certain point mutation in GerAB have the same effect in AGFK germination as was seen for the Δ*gerA* strain, it strengthens our observation that the functionality of the germinosome was affected in strains harboring those point mutations. Note that the Δ*gerA:spe* strain exhibited higher level of germination compared to wt. Albeit out of the scope of the current study, it seems that the spectinomycin resistance marker inserted in the genome could affect AGFK germination. Gao et al. (2022) observed that the presence of an antibiotic resistance gene in cells can by itself have major effects on the bacterial spore proteome.

The GerAB mutant spores constructed in this study without residual GerA showed slower germination kinetics when triggered by AGFK (Figures 3B, D, 4B). This observation was unexpected since AGFK is sensed collaboratively by GerB and GerK, which are not known to need involvement of GerA (Moir et al., 2002). Ultimately, the deletion of the gerA operon also led to a compromised germination in response to AGFK compared to a wt strain (Figure 6). This result was different when gerA: spe was used (Gao et al., 2022), as there was no delayed germination observed when spores were triggered with AGFK. This result indicated a crosstalk between GerA GR and GerB/GerK GR, since the absence of the GerA GR altered the function of GerB and GerK GR. These observations are in agreement with the current understanding of germinosome function where all three GRs in *B. subtilis* spores form protein complexes in germinosomes (Griffiths et al., 2011; Yi et al., 2011) and may exchange GR subunits (Amon et al., 2021). Also, the GerA GR is stimulated via D-glucose binding to GerK, although D-glucose does not trigger spore germination alone (Yi et al., 2011), indicating structural, and even functional interdependency. Taking this even further, the structural integrity of germinosome may require the presence of all three GRs. Our observations provide evidence that the triad is structurally important in the assembly of GerAB and suggest that GerAB structure has an influence on the function of the entire germinosome.

## 3 Discussion

In this study, the function of GerAB was studied by both SMD simulations and mutagenesis. We identified Y97, L199 and F342 forming a barrier to water passage, and therefore investigated their role experimentally. Using GerAB mutagenesis followed by germination assays and western blotting, we found that Y97, L199, and F342 are critical for GerA GR stability and provided more evidence for the dependence between different germination receptors.

However, only Y97A mutant spores could form a functional GerA complex, and thus the role of the triad in water crossing GerAB remain unconfirmed. At the same time, the identification of key residues in GerAB here is mainly based on the analysis of a limited number of simulation trajectories. Note that we present the SMD simulation as an exploration of the mechanisms of water crossing through GerAB, rather than aiming to compute a potential of mean force for a single water molecule going through the protein (Noh and Notman, 2020; Afshinpour et al., 2023).

The current understanding of germination protein function is mostly based on crystallography or static structural prediction (Li et al., 2019; Gao et al., 2023). Here, we aimed to understand GerAB function by exploring its dynamics. From a broader perspective, GerAB belongs to the LeuT-fold transporters superfamily (Jack et al., 2000; Blinker et al., 2021; Alamo et al., 2022). Their function has been well-known as nutrient sensing/transportation (Alamo et al., 2022). Recently, MD studies highlighted the dual functionality of some LeuT transporter proteins as both nutrient sensors and water channels, including vSGLT (Choe et al., 2011) and GkApcT (Afshinpour et al., 2023). GerAB represents germinant receptor B subunit in *Bacillales* and *Clostridiales* spore formers, has its nutrient sensing function better understood than its water channel function (Moir et al., 2002; Christie and Setlow, 2020; Artzi et al., 2021). The dual functionality of LeuT transporters, if also works in GerAB and in other spore-forming species at their GR B subunits, could deepen the understanding of the germination process dynamically. Furthermore, by implementing SMD on GerAB and identifying key residues, this study lays the groundwork for future protein dynamic studies of other membrane-embedded receptor proteins beyond germinant receptors. It also offers a framework for coupling *in silico* work and *in vivo* work for microbiology study not limited by bacterial spores.

## 4 Materials and methods

### 4.1 Simulations and analysis

One 100 ns Molecular Dynamics (MD) simulation of a protein structure model based on *Methanocaldococcus jannaschii* ApcT (PDB: 3GI8) (Shaffer et al., 2009) generated by the structure prediction tool raptorX (Källberg et al., 2012) was conducted using the same starting structure and settings as those described by Blinker et al. (2021). The simulation was performed with GROMACS 2020.4 (Abraham et al., 2015), using the CHARMM36 (Aguayo et al., 2012) forcefield. In a cubic periodic box with starting dimensions of 10 by 10 by 11 nm, the system was solvated by TIP3P water molecules (Mark and Nilsson, 2001). The simulation was maintained at a temperature of 298 K using the Bussi velocity rescaling thermostat (Bussi et al., 2007) and at constant pressure of 1.0 bar with the Parrinello-Rahman barostat (Parrinello and Rahman, 1981; Nosé and Klein, 1983). Long-range electrostatic interactions were treated using the Particle Mesh Edward (Darden et al., 1993) method with a maximum grid spacing at 1.6 Å. For short-range electrostatic and Van-der-Waals interactions, the cutoff radius was set to 12 Å. The production run lasted 100 ns with a timestep of 2 fs, with coordinates saved every 2 ps. The term “(free) MD simulation” refers to the MD simulation production run conducted for this research.

Steered Molecular Dynamics (SMD) simulations were performed to pull a water molecule through the GerAB protein. To extract a suitable starting structure from the MD simulation trajectory, the protein was first positioned at the center of the simulation box with its center of mass between 4.76 nm and 4.85 nm along the x- and y-coordinates, and membrane boundaries at 3.0 nm < z < 7.0 nm. The entrance of the water was defined within the region 3.5 nm < x < 6.5 nm, 3.5 nm < y < 6.5 nm, and 4.6 nm < z < 5.0 nm. Ten frames, each containing a single water molecule within the defined entrance region, were extracted as the starting configurations for Steered Molecular Dynamics (SMD) simulations. The selected water molecule was then subjected to the pulling force during the simulation. The steered MD simulations were conducted using PLUMED 2.7.0 (Bonomi et al., 2009, 2019; Tribello et al., 2014) embedded with GROMACS 2020.4, where the steering collective variable (CV) was the position in the z-direction of the oxygen atom of the pulled water molecule. In all SMD simulations, the selected water molecule was pulled 4 nm over 100 ns along the z-coordinate toward the inside of the inner membrane (IM), following the direction of water intake during germination. Over the 100 ns simulation, a moving harmonic potential kept the water oxygen constrained to z with a force constant κ = 10000 kJ mol^–1^ nm^–2^. The harmonic restraint moved at a constant velocity of 0.04 nm/ns. In addition, the water oxygen atom was constrained in the x and y direction with a harmonic potential with a force constant κ = 10 kJ mol^–1^ nm^–2^, to keep the water molecule inside the protein. The SMD simulations were performed with the same settings as the MD simulation. The work performed to constrain the water molecule along the z coordinate in the SMD simulations was computed as the cumulative force applied to the water molecule over the simulation time. In total, 10 SMD runs were performed. Visual inspection of the simulation trajectories was conducted using Visual Molecular Dynamics (VMD) 1.9.4a53 version (Humphrey et al., 1996). For the 10 starting frames of the SMD simulations, protein contact analysis was carried out via Contact Map Explorer^1^ built on the MDTraj library (McGibbon et al., 2015). First, the minimum distances between all residue pairs in the protein were determined. Two residues are counted as a contact if their minimal distance was 7Å or less. For the three residues in the triad, contacts were counted. Then, a more detailed analysis of the contacts formed by the triad residues was performed by RING, which identifies hydrogen bonds, van der Waals interactions and π – π stacking between residues (Conte et al., 2023).

### 4.2 Mutagenesis

All strains were derived from *B. subtilis* PY79. The *gerA* operon was cloned by PCR, inserted in plasmid vector pUC19 with the Gibson Assembly Master Mix kit (New England BioLabs, NEB # E2611S). Mutagenesis was carried out with the QuikChange Lightning Site-Directed Mutagenesis Kit (Agilent Technologies, Cat # 210518-5). Mutant gerAB sequences were integrated into the original gerAB locus by double crossover along with an erythromycin (erm) resistance cassette. The precision of the mutagenesis in the gerAB locus was confirmed by Sanger sequencing of all mutants. Strains involved in this study are listed in Table 1.

**TABLE 1:** Strains used in this study.

Strain	Strain	Source
gerAB Wild Type (wt)	*Bacillus subtilis* PY79	Lab stock
gerAB Y97A:erm	*Bacillus subtilis* PY79	Constructed in this study
gerAB L199A:erm	*Bacillus subtilis* PY79	Constructed in this study
gerAB F342A:erm	*Bacillus subtilis* PY79	Constructed in this study
gerAB triA:erm	*Bacillus subtilis* PY79	Constructed in this study
gerAB Wild Type (wt)	*Bacillus subtilis* PS832 (Paidhungat and Setlow, 2000)	Prof. Peter Setlow lab, UConn Health
ΔgerA:spe	*Bacillus subtilis* PS832 (Paidhungat and Setlow, 2000)	Prof. Peter Setlow lab, UConn Health
*ΔgerA	*Bacillus subtilis* PS832	Prof. Peter Setlow lab, UConn Health

*Removal of antibiotic resistance gene cassette was done in the lab of Prof. Peter Setlow, UConn Health, unpublished results.

### 4.3 Sporulation and spore purification

Bacterial strains were streaked onto LB agar plates with appropriate antibiotics and incubated overnight to avoid stationary phase; a single colony was inoculated into liquid LB with antibiotics. Once OD_600_ reached 1.0-2.0, 200 μL liquid culture was spread onto 2xSG agar plates (Difco Nutrient Broth 16 g/L, KCl 26 mM, MgSO_4_ 2 mM, MnCl_2_ 0.1 mM, FeSO_4_ 1.08 μM, Ca (NO_3_)_2_ 1 mM, Glucose 5.5 mM, Agar 15 g/L) without antibiotics. Plates were incubated upside down in plastic bags at 37 °C for 2–5 days to allow sporulation, monitored by phase-contrast microscopy. After sporulation, the plates were dried on the benchtop for approximately 2 days to allow cell lysis. Spores were scraped from the plates and transferred to 50 mL centrifuge tubes with cold MQ water. The spore suspensions were then sonicated for 1 min at full power, cooled on ice, and centrifuged at ∼8,000 rpm for 20 min to remove debris. The purity of spore preparation was checked with phase contrast microscopy.

### 4.4 Germination assay with phase contrast microscopy

Purified phase-bright spores at OD_600_ of 5 in Milli-Q water (Milli-Q ultrapure water, resistivity ≥18.2 MΩ⋅cm, Millipore, Billerica, MA, USA) were heat-activated at 70 °C for 30 min followed by 20 min incubation on ice. Before the addition of the germinants, the germinant solutions were kept on ice. A final concentration of 1 mM L-alanine or 10 mM AGFK solution (10 mM each of L-asparagine, D-glucose, D-fructose and KCl) (Cabrera-Martinez et al., 2003; Artzi et al., 2021) was added prior to slide preparation and imaging according to Wen et al. (2019). In essence, to prepare a slide for wide field imaging, slides and two types of coverslips (size 22 mm round and size 22 mm by 30 mm rectangular) were cleaned with 70% EtOH and air dried in a vertical position minimizing dust collection. Two rectangular coverslips were prewarmed for several seconds on a 70 °C heating block, 65 μl 2% agar was deposited on top of one coverslip, while the other coverslip was placed on top of it, spreading the agar in between. The agar patch was dried for approximately 10 min, after which the coverslips were slid off each other. The agar patch was cut into a 1 × 1 cm section to which 0.4 μl of purified spore suspension with germinant added. The patch was transferred onto a round coverslip by placing it onto the patch and sliding it off. A G-frame was stuck onto the air-dried slide, onto which the coverslip was placed, closing all corners of the frame, and completing the slide for microscopy. For wide field time-lapse experiments, we used a Nikon Eclipse Ti equipped with a Nikon Ti Ph3 phase contrast condenser. Connected to it were a Nikon Plan Apo Plan Apo λ Oil Ph3 DM lens (100×, NA = 1.49, *T* = 23 °C), a Lumencor Spectra Multilaser (470 nm, 555 nm) using Lambda 10-B filter blocks, a NIDAQ Lumencor shutter, a Ti XY-and Z drive, and a Hamamatsu C11440-22C camera. All hardware was connected to a computer running NIS-Elements AR 4.50.00 (Build 1117) Patch 03. Before every use the condenser was adjusted to the optimal setting bringing the pixel size to 0.07 μm. A 100-min time lapse video was recorded for every slide with a 30 s interval between frames. The slide chamber was kept at 37 °C, and single-cell germination was analyzed with SporeTrackerX (Omardien et al., 2018). The percentages of germinated spores at the end of the time-lapsed session were defined as germination efficiency.

### 4.5 Germination assay with optical density

Purified phase-bright spores, normalized to an OD_600_ of 1.2 in 25 mM HEPES buffer (pH 7.4), were heat-activated at 70 °C for 30 min, followed by incubation on ice for 20 min. 100 μL of the heat-activated spores were added, and germinant solutions were dispensed into a 96-well plate to a final concentration of 1 mM for L-alanine and 10 mM for AGFK. The OD_600_ was monitored every 2.5 min for 100 min using a plate reader, with the plate maintained at 37 °C and agitated between measurements. The percentage of OD drop is measured against the initial OD. Every experiment was repeated 3 times and the average OD drop for each condition is shown.

### 4.6 Germination assay with DPA release

CaDPA release was measured using a fluorescence plate reader following the method of Yi and Setlow (2010). Spores, at an OD_600_ of 0.5, were germinated with 1 mM L-alanine in 25 mM K-HEPES buffer (pH 7.4) at 45 °C without prior heat activation. At various time points, 190 μL of the sample was mixed with 10 μL of 1 mM TbCl_3_, and relative fluorescent units (RFU) were recorded using a plate reader.

### 4.7 SDS-PAGE and immunoblotting

To prepare samples for western blotting, the spores were first decoated and then lysed as follows. 50 ODs of spores were pelleted by centrifugation. The pelleted spores were resuspended in 1 mL TUDSE buffer (8 M Urea, 50 mM Tris-HCl, pH 8.0, 1% SDS, 50 mM DTT, 10 mM EDTA) and incubated for 45 min at 37 °C, centrifuged (3 min, max rpm, room temperature) and resuspended in 1 mL TUDS (8 M Urea, 50 mM Tris-HCl, pH 8.0, 1% SDS) buffer. The suspension was then incubated for another 45 min at 37 °C, centrifuged (3 min, max rpm, room temperature) and then washed six times by centrifugation (3 min, max rpm, room temperature) and then all suspended in 1 mL TEN buffer (10 mM Tris-HCl pH 8, 10 mM EDTA, 150 mM NaCl). The final decoated spores were resuspended in 1mL water if samples were not immediately given further treatment. 50 OD of the decoated spore preparation was treated with 1 mg lysozyme in 0.5 mL TEP buffer (50 mM Tris-HCl pH 7.4, 5 mM EDTA) containing 1 mM phenylmethylsulfonyl fluoride (PMSF), 1 μg RNase, 1 μg DNase I, and 20 μg of MgCl_2_ at 37 °C for 6–8 min, and then put on ice for 20 min. Glass disruptor beads (0.10–0.18 mm, 100 mg) were added to each sample and spores were disrupted with three bursts of sonication (micro probe, medium power for 10 s, and placed on ice for 30 s between bursts). Following the final sonication, samples were allowed to settle for 15 s and 100 μl of the upper liquid was withdrawn and added to 100 μl 2× Laemmli sample buffer (BIO-RAD cat. #1610737) containing 5% (v/v) 2-mercaptoethanol and 1 mM MgCl_2_ and incubated at 90 °C–95 °C for 3 min. This was saved as the total lysate and ready to run on SDS-PAGE. 20 μg of total protein was loaded onto each lane of SDS-PAGE on a 10% acrylamide gel (BIO-RAD cat. #4561034) and run 45 min at 60 V followed by 45 min at 110 V. After SDS-PAGE, proteins on the gel were transferred to a 0.22 μm PVDF membrane. The membrane was then blocked for 30 min by 2.5% low fat milk in TBST buffer (20 mM Tris, 150 mM NaCl, 0.1% Tween 20) and incubated with 1:3000 anti-GerAA antibody overnight (Ramirez-Peralta et al., 2012). After washing three times with TBST, the membrane was incubated with 1:2500 diluted goat anti rabbit HRP antibody (BIO-RAD cat. #1706515) for an hour before visualization.

## Data Availability

The Input files for the SMD simulations and representative large-field time-lapse videos are available on FigShare at https://doi.org/10.6084/m9.figshare.27225249.v1 and https://doi.org/10.6084/m9.figshare.27226959.v1, respectively.

## Footnotes

1
https://github.com/dwhswenson/contact_map

## References

[B1] Abraham M. J. Murtola T. Schulz R. Páll S. Smith J. C. Hess B. (2015). GROMACS: High performance molecular simulations through multi-level parallelism from laptops to supercomputers. *SoftwareX* 1 19–25. 10.1016/j.softx.2015.06.001

[B2] Afshinpour M. Parsi P. Mahdiuni H. (2023). Investigation of molecular details of a bacterial cationic amino acid transporter (GkApcT) during arginine transportation using molecular dynamics simulation and umbrella sampling techniques. *J. Mol. Model.* 29:260. 10.1007/s00894-023-05670-w 37479900

[B3] Aguayo D. González-Nilo F. D. Chipot C. (2012). Insight into the properties of cardiolipin containing bilayers from molecular dynamics simulations, using a hybrid all-atom/united-atom force field. *J. Chem. Theory Comput.* 8 1765–1773. 10.1021/ct200849k 26593668

[B4] Alamo D. Meiler J. Mchaourab H. S. (2022). Principles of alternating access in LeuT-fold transporters: Commonalities and divergences. *J. Mol. Biol.* 434:167746. 10.1016/j.jmb.2022.167746 35843285

[B5] Amon J. D. Artzi L. Rudner D. Z. (2021). Genetic evidence for signal transduction within the *Bacillus subtilis* GerA germinant receptor. *J. Bacteriol.* 204:e00470-21. 10.1128/jb.00470-21 34780301 PMC8846391

[B6] Artzi L. Alon A. Brock K. P. Green A. G. Tam A. Ramírez-Guadiana F. H. (2021). Dormant spores sense amino acids through the B subunits of their germination receptors. *Nat. Commun.* 12:6842. 10.1038/s41467-021-27235-2 34824238 PMC8617281

[B7] Blinker S. Vreede J. Setlow P. Brul S. (2021). Predicting the structure and dynamics of membrane protein GerAB from *Bacillus subtilis*. *Int. J. Mol. Sci.* 22:3793. 10.3390/ijms22073793 33917581 PMC8038838

[B8] Bonomi M. Branduardi D. Bussi G. Camilloni C. Provasi D. Raiteri P. (2009). PLUMED: A portable plugin for free-energy calculations with molecular dynamics. *arXiv [Preprint]* 10.48550/arxiv.0902.0874

[B9] Bonomi M. Bussi G. Camilloni C. Tribello G. A. Banáš P. Barducci A. (2019). Promoting transparency and reproducibility in enhanced molecular simulations. *Nat. Methods* 16 670–673. 10.1038/s41592-019-0506-8 31363226

[B10] Bussi G. Donadio D. Parrinello M. (2007). Canonical sampling through velocity rescaling. *J. Chem. Phys.* 126:014101. 10.1063/1.2408420 17212484

[B11] Cabrera-Martinez R.-M. Tovar-Rojo F. Vepachedu V. R. Setlow P. (2003). Effects of overexpression of nutrient receptors on germination of spores of Bacillus subtilis. *J. Bacteriol.* 185 2457–2464. 10.1128/jb.185.8.2457-2464.2003 12670969 PMC152624

[B12] Choe S. Rosenberg J. M. Abramson J. Wright E. M. Grabe M. (2011). Water permeation through the sodium-dependent galactose cotransporter vSGLT. *Biophys. J.* 100:248a. 10.1016/j.bpj.2010.12.1572 PMC3042592 20923633

[B13] Christie G. Setlow P. (2020). Bacillus spore germination: Knowns, unknowns and what we need to learn. *Cell Signal* 74:109729. 10.1016/j.cellsig.2020.109729 32721540

[B14] Conte A. D. Monzon A. M. Clementel D. Camagni G. F. Minervini G. Tosatto S. C. E. (2023). RING-PyMOL: Residue interaction networks of structural ensembles and molecular dynamics. *Bioinformatics* 39:btad260. 10.1093/bioinformatics/btad260 37079739 PMC10159649

[B15] Cooper G. R. Moir A. (2011). Amino acid residues in the gerab protein important in the function and assembly of the alanine spore germination receptor of *Bacillus subtilis* 168. *J. Bacteriol.* 193 2261–2267. 10.1128/jb.01397-10 21378181 PMC3133103

[B16] Darden T. York D. Pedersen L. (1993). Particle mesh Ewald: An N ⋅ log(N) method for Ewald sums in large systems. *J. Chem. Phys.* 98 10089–10092. 10.1063/1.464397

[B17] Gao X. Swarge B. N. Roseboom W. Wang Y. Dekker H. L. Setlow P. (2022). Changes in the spore proteome of *Bacillus cereus* in response to introduction of plasmids. *Microorganisms* 10:1695. 10.3390/microorganisms10091695 36144297 PMC9503168

[B18] Gao Y. Amon J. D. Artzi L. Ramírez-Guadiana F. H. Brock K. P. Cofsky J. C. (2023). Bacterial spore germination receptors are nutrient-gated ion channels. *Science* 380 387–391. 10.1126/science.adg9829 37104613 PMC11154005

[B19] Griffiths K. K. Zhang J. Cowan A. E. Yu J. Setlow P. (2011). Germination proteins in the inner membrane of dormant *Bacillus subtilis* spores colocalize in a discrete cluster. *Mol. Microbiol.* 81 1061–1077. 10.1111/j.1365-2958.2011.07753.x 21696470 PMC7959159

[B20] Humphrey W. Dalke A. Schulten K. (1996). VMD: Visual molecular dynamics. *J. Mol. Graph.* 14 27–28. 10.1016/0263-7855(96)00018-5 8744570

[B21] Jack D. L. Paulsen I. T. Saier M. H. (2000). The amino acid/polyamine/organocation (APC) superfamily of transporters specific for amino acids, polyamines and organocations. *Microbiology* 146 1797–1814. 10.1099/00221287-146-8-1797 10931886

[B22] Jumper J. Evans R. Pritzel A. Green T. Figurnov M. Ronneberger O. (2021). Highly accurate protein structure prediction with AlphaFold. *Nature* 596 583–589. 10.1038/s41586-021-03819-2 34265844 PMC8371605

[B23] Källberg M. Wang H. Wang S. Peng J. Wang Z. Lu H. (2012). Template-based protein structure modeling using the RaptorX web server. *Nat. Protoc.* 7 1511–1522. 10.1038/nprot.2012.085 22814390 PMC4730388

[B24] Knudsen S. M. Cermak N. Delgado F. F. Setlow B. Setlow P. Manalis S. R. (2015). Water and small-molecule permeation of dormant *Bacillus subtilis* spores. *J. Bacteriol.* 198 168–177. 10.1128/jb.00435-15 26483518 PMC4686191

[B25] Li Y. Jin K. Perez-Valdespino A. Federkiewicz K. Davis A. Maciejewski M. W. (2019). Structural and functional analyses of the N-terminal domain of the A subunit of a *Bacillus megaterium* spore germinant receptor. *Proc. Natl. Acad. Sci.* 116 11470–11479. 10.1073/pnas.1903675116 31113879 PMC6561283

[B26] Madeira F. Madhusoodanan N. Lee J. Eusebi A. Niewielska A. Tivey A. R. N. (2024). The EMBL-EBI Job dispatcher sequence analysis tools framework in 2024. *Nucleic Acids Res.* 52 W521–W525. 10.1093/nar/gkae241 38597606 PMC11223882

[B27] Maginn E. J. (2007). Atomistic simulation of the thermodynamic and transport properties of ionic liquids. *Acc. Chem. Res.* 40 1200–1207. 10.1021/ar700163c 17953449

[B28] Mark P. Nilsson L. (2001). Structure and dynamics of the TIP3P, SPC, and SPC/E water models at 298 K. *J. Phys. Chem. A* 105 9954–9960. 10.1021/jp003020w

[B29] McGibbon R. T. Beauchamp K. A. Harrigan M. P. Klein C. Swails J. M. Hernández C. X. (2015). MDTraj: A modern open library for the analysis of molecular dynamics trajectories. *Biophys. J.* 109 1528–1532. 10.1016/j.bpj.2015.08.015 26488642 PMC4623899

[B30] Moir A. Corfe B. M. Behravan J. (2002). Spore germination. *Cell. Mol. Life Sci. CMLS* 59, 403–409. 10.1007/s00018-002-8432-8 11964118 PMC11337513

[B31] Mongkolthanaruk W. Cooper G. R. Mawer J. S. P. Allan R. N. Moir A. (2011). Effect of amino acid substitutions in the GerAA protein on the function of the alanine-responsive germinant receptor of *Bacillus subtilis* spores. *J. Bacteriol.* 193 2268–2275. 10.1128/jb.01398-10 21378197 PMC3133101

[B32] Noh S. Y. Notman R. (2020). Comparison of umbrella sampling and steered molecular dynamics methods for computing free energy profiles of aromatic substrates through phospholipid bilayers. *J. Chem. Phys.* 153:034115. 10.1063/5.0016114 32716163

[B33] Nosé S. Klein M. L. (1983). Constant pressure molecular dynamics for molecular systems. *Mol. Phys.* 50 1055–1076. 10.1080/00268978300102851

[B34] Omardien S. Beek A. T. Vischer N. Montijn R. Schuren F. Brul S. (2018). Evaluating novel synthetic compounds active against *Bacillus subtilis* and *Bacillus cereus* spores using live imaging with SporeTrackerX. *Sci. Rep.* 8:9128. 10.1038/s41598-018-27529-4 29904100 PMC6002552

[B35] Paidhungat M. Setlow P. (2000). Role of ger proteins in nutrient and nonnutrient triggering of spore germination in *Bacillus subtilis*. *J. Bacteriol.* 182 2513–2519. 10.1128/jb.182.9.2513-2519.2000 10762253 PMC111315

[B36] Parrinello M. Rahman A. (1981). Polymorphic transitions in single crystals: A new molecular dynamics method. *J. Appl. Phys.* 52 7182–7190. 10.1063/1.328693

[B37] Ramirez-Peralta A. Zhang P. Li Y. Setlow P. (2012). Effects of sporulation conditions on the germination and germination protein levels of *Bacillus subtilis* spores. *Appl. Environ. Microbiol.* 78 2689–2697. 10.1128/aem.07908-11 22327596 PMC3318806

[B38] Shaffer P. L. Goehring A. Shankaranarayanan A. Gouaux E. (2009). Structure and mechanism of a Na+-independent amino acid transporter. *Science* 325 1010–1014. 10.1126/science.1176088 19608859 PMC2851542

[B39] Smart O. S. Neduvelil J. G. Wang X. Wallace B. A. Sansom M. S. P. (1996). HOLE: A program for the analysis of the pore dimensions of ion channel structural models. *J. Mol. Graph.* 14 354–360. 10.1016/s0263-7855(97)00009-x 9195488

[B40] Sunde E. P. Setlow P. Hederstedt L. Halle B. (2009). The physical state of water in bacterial spores. *Proc. Natl. Acad. Sci.* 106 19334–19339. 10.1073/pnas.0908712106 19892742 PMC2780810

[B41] Tribello G. A. Bonomi M. Branduardi D. Camilloni C. Bussi G. (2014). PLUMED 2: New feathers for an old bird. *Comput. Phys. Commun.* 185 604–613. 10.1016/j.cpc.2013.09.018

[B42] Wen J. Pasman R. Manders E. M. Setlow P. Brul S. (2019). Visualization of germinosomes and the inner membrane in *Bacillus subtilis* spores. *J. Vis. Exp*. e59388. 10.3791/59388 31033949

[B43] Wilson M. J. Carlson P. E. Janes B. K. Hanna P. C. (2012). Membrane Topology of the *Bacillus anthracis* GerH germinant receptor proteins. *J. Bacteriol.* 194 1369–1377. 10.1128/jb.06538-11 22178966 PMC3294866

[B44] Yi X. Setlow P. (2010). Studies of the commitment step in the germination of spores of *bacillus* species. *J. Bacteriol.* 192 3424–3433. 10.1128/jb.00326-10 20435722 PMC2897682

[B45] Yi X. Liu J. Faeder J. R. Setlow P. (2011). Synergism between different germinant receptors in the germination of *bacillus subtilis* spores. *J. Bacteriol.* 193 4664–4671. 10.1128/jb.05343-11 21725007 PMC3165675

[B46] Zuberi A. R. Moir A. Feavers I. M. (1987). The nucleotide sequence and gene organization of the gerA spore germination operon of *Bacillus subtilis* 168. *Gene* 51 1–11. 10.1016/0378-1119(87)90468-9 3110007

