# Supplementary material for: GerAB residues predicted to interfere with water passage based on steered molecular dynamics are key to germinosome functionality
Source: Front Microbiol. 2025 Sep 24;16:1656964. doi: 10.3389/fmicb.2025.1656964 (PMC12504216; doi:10.3389/fmicb.2025.1656964)
Supplement: Supplementary file 1 [file Data_Sheet_1.pdf]

Supplementary information

```
GerRB|AAP07773.1      MNTMNKTKTVSPYFAFILLHSLQIGIGILGYQRVIL-KNAGYDAWISLIIAGIATHIVLF      59
GerKB|CAB12180.1      ----MEKARISIRQLFVMIIIFELGSSLLITPGSMA----GRDAWIAVLLGCAIGLFLFY      52
GerVB|ADE72615.1      ----MEKAKISASQLFILMVLFELGSSLLVPIAMDA----KQDAWLAIKIGMVGSFVLFL      52
GerAB|KZD90227.1      MSQKQTPLKLNTFQGISIVANTMLGAGLLTLPRALTTKANTPDGWITLILEGFIFIFFIY      60
GerBB|CAB15598.1      --MRKSEHKLTFMQTLIMISSTLIGAGVLTLPRSAA-ETGSPSGWLMILLQGVIFIIIVL      57
                               :.    : ::    :* .:*             ..*: :::      ...

GerRB|AAP07773.1      CMLKMLEKD--GDLISIHTTTFGKWIGSIFSVIFTLYCLLFCLTVLRTYMEVIQVWIFPT      117
GerKB|CAB12180.1      LYQGIYQCYPNSSPKEYMDDMLGTKLSWLFSFLYILYFAYIAARVLRDFGEMLLTFAYHD      112
GerVB|ADE72615.1      VYHKLHSYYPDLLPTEYMQKIMGKGIGTVLAFVYILYFMYDAARVLRDFGAMLLTSAYPE      112
GerAB|KZD90227.1      LNTLIQKKHQYPSLFEYLKEGLGKWIGSIIGLLICGYFLGVASFETRAMAEMVKFFLLER      120
GerBB|CAB15598.1      LFLPFLQKNSGKTLFKLNSIVAGKFIGFLLNLYICLYFIGIVCFQARILGEVVGFFLLKN      117
                          : .        .      *. :. :: .    *         *    ::

GerRB|AAP07773.1      IKLWKLTLMFLLVTYYIIKGGFRSVTGICFWGIVL---PMFVVFFLVYPMKYAHFRNILP      174
GerKB|CAB12180.1      TPIIIVNALLMVVSIYAVRKGIEVLARAAELLFGAMYLLGAIGLVLIIVSGTIDPHNLKP      172
GerVB|ADE72615.1      TPLFIAHTLLMLVVIYTIRKGIEVVARSGEILFIFIYIFAIMGFVLIVCSGLIEFTNLQP      172
GerAB|KZD90227.1      TPIQVIILTFICCGIYLIVGGLSDVSRLFLFYLTV--TIIILLIVFGISFKIFDINNLRP      178
GerBB|CAB15598.1      TRMAVVVFIFLAVAIYHVGGGVYSIAKVYAYIFPI--TLIIFMMLLMFSFRLFQLDFIRP      175
                        :      ::    * :  *.  ::      :        . :.:       .   : *

GerRB|AAP07773.1      IFTHSPFDILVSAKQSALEFLGFETILI--FYPFIEKGKSLKRWAHAGIVFSTLIYVVLA      232
GerKB|CAB12180.1      VLANGISPVLHSVFTQTMYVPFGEVVLFVMIFPNLNDRKDVKKMGMIAMAISGLIVALTV      232
GerVB|ADE72615.1      VLEEGVLPVLKVAFTQTIYFPFAEAMVFTMILPYLKDQKKAKMTMLCATGLSGINLTITM      232
GerAB|KZD90227.1      VLGEGLGPIANSLTVVSISFLGMEVMLF--LPEHMKKKKYTFRYASLGFLIPIILYILTY      236
GerBB|CAB15598.1      VFEGGYQSFFSLFPKTLLYFSGFEIIFY--LVPFMRDPKQVKKAVALGIATSTLFYSITL      233
                      ::  .   .        : .   *  :.  :   :.. *        .     :   :

GerRB|AAP07773.1      IVSFMYYSEGQLNHTIWPTLTMLKIIKVP--FIQRFEYIIIFVWFLIILPNLCLTIWSSC      290
GerKB|CAB12180.1      AINISVLDVDLTLRSQFPLLSTIQTIKVE-EFLDRLDVFFMLALIIGGFFKVSLYLYATV      291
GerVB|ADE72615.1      LINISVLGVDLTSRSQFPLLSTVQSIQVA-DFLERLDVFFMLALVIGGFIKISVLLYAAV      291
GerAB|KZD90227.1      IIVVGALTAPEVKTLIWPTISLFQSFELKGIFIERFESFLLVVWIIQFFTTFVIYGYFAA      296
GerBB|CAB15598.1      LIVIGCMTVAEAKTVTWPTISLIHALEVPGIFIERFDLFLQLTWTAQQFACMLGSFKGAH      293
                       : .            :* :: .:  :::  *::*:: :: ..     :  .      :

GerRB|AAP07773.1      QTMKRSFHISFKFTLPFFI--FI---VFTASLFFKNRESINALNTVLSQAGLYIVY---A      342
GerKB|CAB12180.1      VGTSTLFKEKNPSQLAYPMGLGILILSITIATNFSEH-----LNEGLNVVPLYIHL----      342
GerVB|ADE72615.1      IGTANLFKIKSPSRLSYPLGFVILFMASTIASNFQEH-----LHEGLKVEMFILHM----      342
GerAB|KZD90227.1      NGLKKTFGLSTKTSMV----------IIGIAVFYFSL-----WPDDANQVMMYSDYLGYI      341
GerBB|CAB15598.1      IGLTEIFHLKNKNNAWLL-------TAMLAATFFITM-----YPKDLNDVFYYGTLLGYA      341
                            *  .                    :  :             .

GerRB|AAP07773.1      YIPILFLVHSLR-----WRFKNQSKKSSTDTP- 369
GerKB|CAB12180.1      --PFQLLFPLFLFIVAVWKKKRREKSKGEEAKK 373
GerVB|ADE72615.1      --PILAIIPSLLLLVAFLKNRKKQRG------- 366
GerAB|KZD90227.1      F-VSLFLLPFILFFIV-ALKRRITAK------- 365
GerBB|CAB15598.1      F-LIVITIPFFVWFLS-WIQKKIGRGQLQ---- 368
                             .  :         :.
```

Figure S1. Clustal Omega multiple sequence alignment of GerAB and homologs from *Bacillus* species, including GerRB from *Bacillus cereus*, and GerAB, GerBB, and GerKB from *Bacillus subtilis*, as well as GerVB from *Bacillus megaterium*. GenBank accession numbers are shown. Key residues identified in this study, Y97, L199, and F342 in GerAB, are highlighted in red. Y97 is fully conserved across the three germinant receptors.

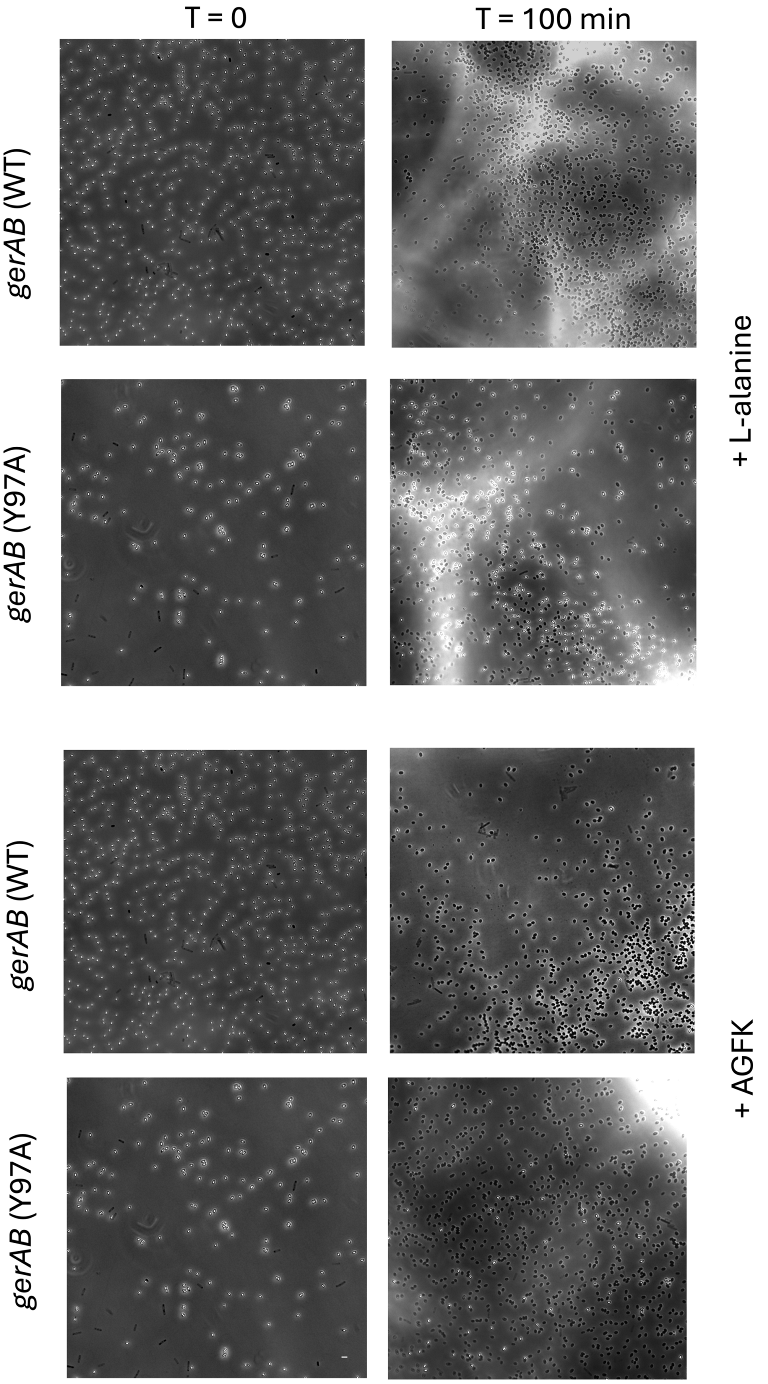

Figure S2. Uncropped microscopy image of representative germination assay of wt (wild-type) and Y97A spores before and after 100 min incubation with L-alanine or AGFK (L-asparagine, 1D-glucose, D-fructose and K^+ ions), respectively. Scale bar, 2 μm.

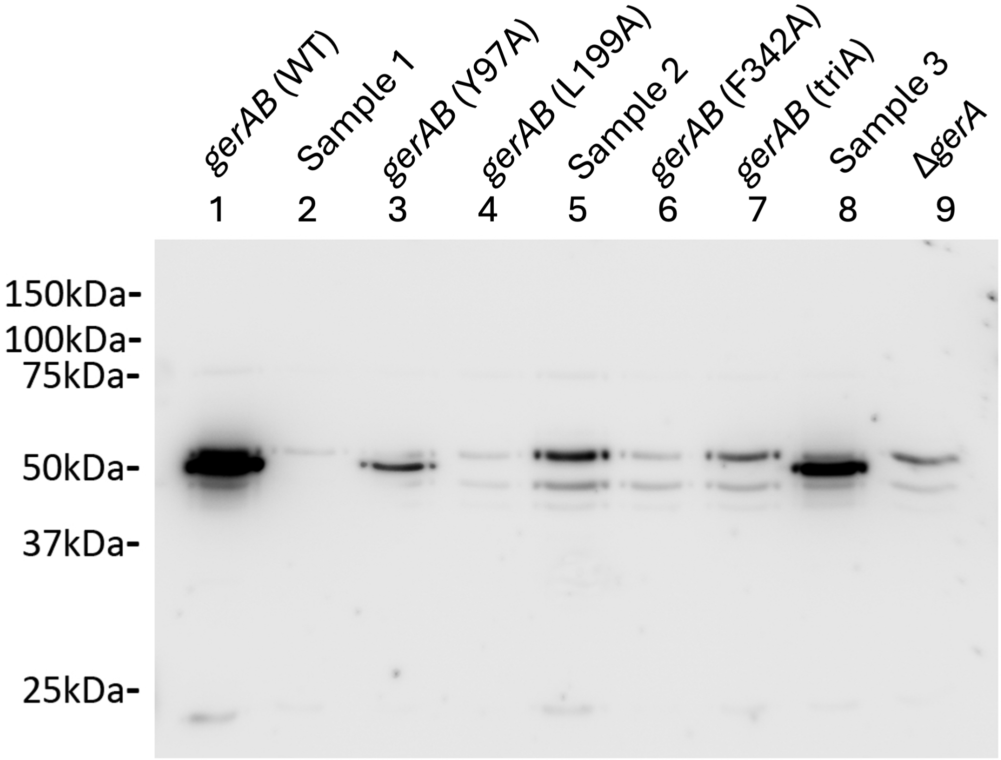

Figure S3. Uncropped western blot of *B. subtilis* PY79 wt and mutant spore proteins with GerAA antibody. Sample 1, 2 and 3 were removed from Figure 5 in main text, as they are irrelevant from the current study.

SMD Run Number	1 (Δw_{min})	2	3	4	5	6 (Δw_{max})	7	8	9	10
**π - π stacking Y97**	1	1	1	1	1	0	0	0	0	0
**π - π stacking F342**	1	1	2	1	2	1	2	1	1	1
**Residue contact count Y97**	20	20	24	23	23	21	24	23	19	24
**Residue contact count L199**	27	25	22	28	25	23	25	22	23	22
**Residue contact count F342**	22	21	21	20	22	22	21	23	21	19

Table S1. Contact count of Y97, L199 and F342 within GerAB for start structures each SMD run. Runs with Δw_{min} and Δw_{max} were labeled. F342 forms more π – π stacking than Y97 in eight runs while Y97 forms π – π stacking with only F342 in five runs. L199 exhibits higher total residue contact than Y97 and F342 in seven starting structures.
